# Supplementary material for: Changes in the Texture and Flavor of Lotus Root after Different Cooking Methods
Source: Foods. 2023 May 16;12(10):2012. doi: 10.3390/foods12102012 (PMC10217657; doi:10.3390/foods12102012)
Supplement: Supplementary file 1 [file foods-12-02012-s001.zip › foods-2326286-supplementary.pdf]

Table S1. Scheme of elution gradient for HPLC - DAD analysis.

Time (min)	Solvent (A %)	Solvent (B %)
0	10	90
10	18	82
15	24	76
21	41	59
21.5	41	59
22	42.2	57.8
23	42.5	57.5
27	58	42
29	59	41
31	59.5	40.5
32	60	40
48	60	40
50	10	90
55	10	90
